# Cats and Allergies

**DOI:** 10.1371/journal.pmed.0020094

**Published:** 2005-03-29

The prevalence of asthma and allergy has risen in all industrialized countries during recent decades, and there is much debate about exposure to pets in early life and later development of asthma and allergy. Some studies have suggested that keeping pets actually protects against later allergy—i.e., that early exposure may somehow modify an individual's immune system to tolerate specific antigens. What might be the mechanism for such protection against allergy? One theory of how allergies arise is that an imbalance in T helper cell subtypes tips the body's immune response towards overreacting to a particular antigen. There is some evidence that early exposure to high natural levels of cat allergens can prevent such an inappropriate immune response. Other researchers have suggested that normally immune responses are kept under control by another group of T cells—regulatory T cells. The two mechanisms may be linked, since exposure to high levels of cat allergens may induce regulatory T cells.

Various attempts to modify aberrant immune responses to specific allergens, such as those to cat dander, have been made. Investigators have treated patients with related molecules, either peptides derived from the allergen itself, or much smaller peptides produced synthetically. Although therapy with peptides seems to reduce allergic responses, the mechanism of the response to treatment has not been clear, in particular, exactly which cells, cell surface markers, and cytokines are involved in modifying the immune response.

**Figure:**
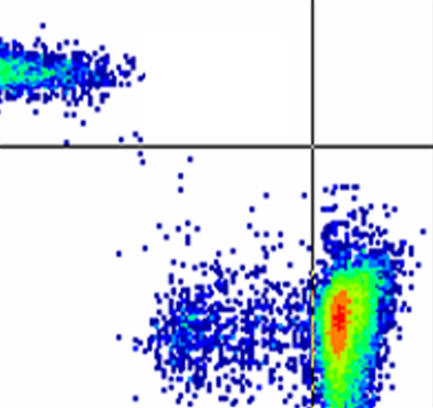
Identifying the T cells that suppress proliferation to allergens after immunotherapy

In a paper in this month's *PLoS Medicine*, Mark Larché and colleagues have attempted to dissect out this pathway in a group of individuals with asthma and allergy to cats. They treated the individuals with short synthetic peptides derived from the sequence of the major cat allergen, *Felis domesticus* allergen 1, and then measured the clinical and immunological response to allergen. They found that treatment with the peptides led to the induction of a population of T cells that were capable of suppressing the proliferation of allergen-reactive T cells in vitro. Peptide treatment also resulted in increased levels of a molecule called CD5 on the surface of blood T cells—CD5 has recently been associated with suppressing T cell sensitivity to stimulation. Finally, the authors found that the degree of suppression was not related to the amount of peptide given to the patients.

Where does this finding leave patients who might wonder about exposure to cats and the development of allergy? The simple answer is that we do not know exactly how exposure to antigen triggers either an immune reaction or tolerance. Once triggered, an immune reaction to a cat may be hard—though not impossible—to reverse, but how or why a specific individual becomes sensitized is as yet far from clear.

